# Water Caltrop (*Trapa quadrispinosa* Roxb.) Husk Improves Oxidative Stress and Postprandial Blood Glucose in Diabetes: Phenolic Profiles, Antioxidant Activities and α-Glycosidase Inhibition of Different Fractions with In Vitro and In Silico Analyses

**DOI:** 10.3390/antiox11101873

**Published:** 2022-09-21

**Authors:** Yuanyue Zhang, Shengbao Cai, Shuang Ma, Shuai Zhao, Junjie Yi, Linyan Zhou

**Affiliations:** Faculty of Food Science and Engineering, Kunming University of Science and Technology, Kunming 650500, China

**Keywords:** antioxidant activity, phenolics, UHPLC-ESI-HRMS/MS, molecular docking, molecular dynamics, α-glycosidase inhibition

## Abstract

The aim of this study was to investigate the phenolic profiles, antioxidant activities and α-glycosidase inhibitory activities of three different phenolic fractions from water caltrop (*Trapa quadrispinosa* Roxb.) husk and to further explore the predominant compounds and their mechanisms on α-glycosidase inhibition by virtual screening and molecular dynamics. A total of 29 substances were identified and quantified in this study. Tannins were the main constituents of water caltrop husk extract. All of the free phenolic (FP), esterified phenolic (EP) and insoluble-bound phenolic (BP) fractions exhibited good antioxidant activities, and the BP had the highest radical scavenging ability with IC_50_ values of 0.82 ± 0.12 μg/mL (ABTS) and 1.15 ± 0.02 μg/mL (DPPH), respectively (*p* < 0.05). However, compared with the EP and BP, the FP showed the strongest inhibition towards the α-glycosidase and the IC_50_ value of FP was 1.43 ± 0.12 μg/mL. The 1,2,6-trigalloylglucose and α-glycosidase complex had better root mean square deviations (RMSD) stability via molecular dynamics simulation study. Results obtained from this study may provide a good potential natural resource for the improvement of oxidative stress injury and blood glucose control in diabetes mellitus, which could expand the use of water caltrop husk and improve its economic value.

## 1. Introduction

Diabetes is a serious chronic disease characterized by hyperglycemia, which is the third leading killer worldwide after cardiovascular disease and cancer [1]. Prolonged hyperglycemia will cause severe oxidative stress damage to the blood vessels, which eventually leads to inflammatory responses, atherosclerosis and other complications of diabetes [1,2]. Therefore, the treatment of diabetes not only needs to control blood glucose, especially the postprandial blood glucose, but also needs to improve the oxidative stress of the body. In current clinical treatment, acarbose can inhibit α-glycosidase activity to prolong intestinal digestion of carbohydrates to control postprandial blood glucose [3]. However, acarbose cannot improve the oxidative stress damage caused by hyperglycemia [4]. Antioxidants used in clinical practice (e.g., vitamin C, vitamin E) have the effect of scavenging free radicals to reduce oxidative damage, but do not directly control blood glucose [5]. The inability to combine glucose-controlling effect and antioxidant activity often results in the need for diabetic patients to take multiple drugs, which brings great physical and economic burden to patients. Therefore, it is important and urgent to find substances that possess both antioxidant activity and glucose-controlling effect.

Phenolics are a kind of important secondary metabolites for plant defense during growth [6]. Numerous previous studies have found that phenolics not only had good antioxidant activity but also possessed strong inhibitory effect towards α–glycosidase [7,8]. Yang et al. found that polyphenols in artichoke (*Cynara Scolymus* L.) have strong antioxidant activity [7]. Moreover, a previous study showed that cyanidin-3-O-glucoside (C3G) could effectively prevent advanced glycosylation end products (AGEs)-induced inflammation and vascular endothelial dysfunction, which partly due to its good antioxidant activity [9], and ferulic acid could also effectively inhibit AGEs-induced inflammatory responses in human umbilical vein endothelial cells (HUVEC, a model system for the study of endothelial cell function) [10]. Yue et al. found that phenolics in *Rheum Tanguticum* Maxim. Ex Balf. (RT) had strong α–glycosidase inhibitory activity, and Jia et al. found that the structures of dietary flavonoids exhibited significant effects on their inhibitory activities towards α–glycosidase [8,9]. In addition, it is well known that phenolics in plants mainly exist in three different forms, namely, free phenolics (FP) (mainly proanthocyanidins and flavonoids), esterified phenolics (EP) (mostly phenolic acids) and insoluble-bound phenolics (BP) (combined with cellulose or proteins in plant tissues). The bioactivities of phenolics are often closely related with their different forms, even in the same plant. Zhou et al. found that the BP fraction in oil palm fruits have the strongest antioxidant and cell protective activities when compared with the FP and EP fraction [11]. Liu et al. found that FB fraction of *Rhus chinensis* Mill. fruits showed the strongest anti-diabetes activity in vitro in comparison with EP and BP fraction [12]. Those studies suggested that phenolics in plants with different forms may serve as good candidate compounds that have a great potential to exert both antioxidant activity and glucose-controlling effect to either improve diabetes, its complications or both. Therefore, many researchers are focusing on phenolic-rich plants to exploit their health-promoting usage for elevating their economic value.

Water caltrop (*Trapa quadrispinosa* Roxb.) is an annual floating aquatic plant commonly cultured in freshwater wetlands, lakes, ponds and slow-flowing rivers, which is widely grown in Asia, Europe and other regions [13,14]. It was found that the fruit husk is rich in phenolics and has analgesic, antiulcer, immune regulation, hypoglycemic and other activities [13,15]. Moreover, there is a history of using the husk as an anti-diabetic medicine in China [16]. However, the phenolic composition and distribution of FP, EP and BP from water caltrop husk, as well as their inhibition of α-glycosidase activity, are rarely studied. In this paper, the phenolic profiles and distribution characteristics of FP, EP and BP in water caltrop husk were qualitatively and quantitatively analyzed, and their antioxidant activities and α-glycosidase inhibitory effects were comparatively evaluated. Moreover, the predominant compounds and their α-glycosidase inhibitory mechanisms were also comprehensively investigated by molecular docking and molecular dynamics simulation. Results obtained from this study may provide some new insights into water caltrop husk as a good potential natural resource for improving oxidative stress injury and blood glucose control in diabetes, which may further promote the economic value of this crop.

## 2. Materials and Methods

### 2.1. Chemical and Reagents

Acetonitrile, formic acid and methanol of mass spectrometry grade were purchased from Merck (Darmstadt, Germany). Furthermore, 2,2′-Azino-bis (3-ethylbenzothiazoline-6-sulfonic acid) (ABTS), 2-diphenyl-1-pi-crylhydrazyl radical (DPPH), α-glycosidase (source: *Saccharaccharides cerevisiae*, ≥10 U/mg protein), p-nitrobenzene-α-D-glucoside and acarbose were purchased from Sigma-Aldrich (Shanghai, China). The other chemical reagents used in the experiments were of analytical grade.

### 2.2. Sample Preparation

Water caltrop was harvested in August 2021 in the Weishan Lake area of Weishan County, Jining City, Shandong Province. Husks were manually peeled and then air dried for two weeks. After that, the dried husks were stored at −20 °C for further experiments. The FP, EP and BP fractions were extracted according to previous methods [11,17]. The dried water caltrop husks were ground with a high-speed grinder (Lingdan LD-T300, Shanghai, China) in order to pass through an 80-mesh sieve and the powder was collected and stored at −20 °C. Then, 30 g of powder was firstly defatted with 150 mL of petroleum ether in an ultrasonic bath (200 W) for 30 min. The defatted water caltrop husk powder was ultrasonically extracted with 150 mL of 70% methanol and 70% acetone (1:1 *v*/*v*) for 30 min, and then, the mixture was filtered, and the filtrate was collected. The process was repeated two times. The filtrate was collected and evaporated by using a rotary evaporator (Hei-VAP, Heidolph, Schwabach, Germany) to remove the organic reagents and the aqueous phase was collected and used to extract the FP and EP. For extracting the FP, the above aqueous phase was first adjusted to pH = 2 by using 6 M hydrochloric acid, which was then extracted with ether and ethyl acetate (1:1 *v*/*v*) six times. The ether-ethyl acetate phase was evaporated by using a rotary evaporator to remove the organic reagents and the FP extract was obtained after lyophilization. For the extraction of the EP, the remaining aqueous phase after FP extraction was supplemented with 4 M sodium hydroxide (1:10 *v*/*v*) and hydrolyzed at room temperature for 4 h. The remaining procedures was similar to that for the extraction of the FP. After extraction of FP and EP, the remaining solid residues were hydrolyzed with 4 M sodium hydroxide (1:10 *v*/*v*) at room temperature for 4 h and filtered. The filtrate was collected and adjusted to pH = 2 with 6 M hydrochloric acid. The acidified solution was defatted with petroleum ether three times, and then extracted with ether and ethyl acetate (1:1 *v*/*v*) by the same method as the above-mentioned procedure. All extracts were lyophilized (Alpha 1-2 LD plus, Christ, Germany) for 48 h, which were finely ground and separately sealed in a ziplock bag. Thereafter, the ziplock bag was place in desiccant and stored at −20 °C. The pH values (PHS-3G, Shanghai, China) of FP, EP and BP at the same concentration as prepared with PBS for the α-glycosidase assay were about 6.80 ± 0.32. All experiments in the current work were conducted by using the same batch of cultured water caltrop from the same origin, and all indicators were determined within one month after sample extraction.

### 2.3. Determination of Total Phenolic Content (TPC)

The TPC of three different fractions of water caltrop husk were determined through the Folin–Ciocalteu reagent method reported by references [7,11,17].

### 2.4. Determination of Total Flavonoid Content (TFC)

The TFC of three different fractions of water caltrop husk were measured by the method reported by references [7,11,17].

### 2.5. Characterization of Phenolics by UHPLC-ESI-HRMS/MS

The phenolic compounds of the three different fractions were identified and quantified by using a Thermo Fisher Ultimate 3000 UHPLC System (Merck, Darmstadt, Germany) equipped with a Q-Exactive Orbitrap mass spectrometer. Phenolic separation was performed by a Poroshell 120 SB-C18 column (2.1 × 100 mm^2^, 2.7 μm, USA) with an injection volume of 1.0 μL, flow rate of 0.2 mL/min and column temperature of 30 °C. Acidified water (0.1% formic acid, phase A) and acetonitrile (phase B) were used as mobile phases in the following gradient: 0–2 min, 5% B; 2–18 min, 5–35% B; 18–20 min, 35–5% B; and 20–22 min, 5% B. Mass spectrum data in the negative mode were recorded. Full MS scan was used with *m*/*z* at 50–1000. The related parameters of MS were measured by the identical method described earlier [9,18].

### 2.6. ABTS Radical Scavenging Activity

The ABTS radical scavenging activities of three different fractions of water caltrop husk were measured by the identical method described earlier [7,11].

### 2.7. DPPH Radical Scavenging Activity

The DPPH radical scavenging activities of three different fractions of water caltrop husk were determined by the same method reported earlier [7,11].

### 2.8. Inhibition of α-Glycosidase

α-Glycosidase inhibitory activity was evaluated according to published methods [19,20]. Briefly, 10 μL of α-glycosidase (200 μg/mL), 10 μL of tested sample and 110 μL of phosphate buffer saline (PBS) were mixed and incubated at 37 °C for 30 min, and then 20 μL of pNPG (2.5 mM) was added and incubated at 37 °C for another 30 min. After incubation, 60 μL of 0.2 mol/L of Na_2_CO_3_ was added to stop the reaction. The reaction mixture without sample was used as the control group. Absorbance values of all mixtures were read at 405 nm by microplate reader. The calculation formula was as follows: α-glycosidase inhibitory rate (%) = (absorbance value of control group—absorbance value of sample group)/absorbance value of control group × 100.

### 2.9. Correlation Analysis

The correlation analysis was performed by the genescloud tools, a free online platform for data analysis (https://www.genescloud.cn (accessed on 5 August 2022)).

### 2.10. Molecular Docking and Molecular Dynamics (MD) Simulation

Autodock Vina software [21] was used for molecular docking research. The 3D crystal structure of α-glycosidase (PDB code: 3A4A) is available for download in the RCSB database (https://www.rcsb.org/pdb (accessed on 13 July 2020)), with PDB code of the crystal structures of α-glycosidase with reference to previous studies [9,19]. Ligand and water were removed from the protein by using PyMol software 2.4 (DeLano, 2002 (Warren Lyford DeLano, San Carlos, CA, USA, Free.)). Compounds of water caltrop husk identified by UHPLC-ESI-HRMS/MS were used as ligands. The structure of each compound was downloaded from Pubchem database (https://pubchem.ncbi.nlm.nih.gov/ (accessed on 13 July 2020)), and the compound IDs are summarized in Table 1. The protein and ligand were converted from PDB to PDBQT by using Autoduck Tools software (ADT, version 1.5.6). The semi-flexible docking process was performed on Autodock vina software (https://vina.scripps.edu/ (accessed on 13 July 2020)) with center box: x = 25.38, y = −2.75 and z = 18.186, with dimensions: 52 × 68 × 62 Å. Discovery Studio 2019 Client (Biovia Co., San Diego, CA, USA) and PyMol software were used to visualize the analysis of the binding force of ligand and protein after molecular docking.

MD simulation was further performed on the basis of molecular docking. The results of MD simulation can explore the differences in stability of three different isomerides of trigalloylglucose and three different isomerides of tetragalloylglucose after forming complexes with α-glycosidase and figure out which is the best one. All MD simulations were performed using GROMACS 19.5 Package (https://manual.gromacs.org/ (accessed on 15 July 2020)) with the Amber ff99SB-ILDN force field. The system was solvated in TIP3P explicit water model with addition of sodium and chloride ions to neutralize the charge and result in a system NaCl concentration of 0.15 M. The steepest descent method was used to minimize energy (less than 1000.0 kJ/mol/nm). After the energy minimization, canonical ensemble simulation (NVT, 2 ns) was performed, followed by isothermal-isobaric simulation (NPT, 1 ns), and the system can process MD (100 ns) at constant temperature and pressure (310.15 K, 1 bar). After the MD, the stability of each trigalloylglucose isomeride combining with α-glycosidase was analyzed by using root mean square deviations (RMSD), root mean square fluctuations (RMSF) and number of hydrogen bonds (number of H-bonds).

### 2.11. Statistical Analysis

All experiments were measured three times, and the experimental data were expressed as the mean ± standard deviation (*n* = 3). One-way ANOVA and Tukey’s test were used for testing the significance of the difference (*p* < 0.05). All analyses were performed by using Origin8.5 software (OriginLab, Northampton, MA, USA).

## 3. Results and Discussion

### 3.1. Identification and Quantification of Phenolic Compounds and TFC and TPC

In previous studies, tannin [15], flavonoids [9], carbohydrates [13] and other chemicals were found in the secondary metabolites of various extracts of water caltrop husk [13,15]. These chemicals are also known as lifetime metabolites of plants, and have been reported with some bioactivities, such as improving diabetes [19], cancer [22,23], gastric ulcer [24], alcoholic fatty liver disease [25], nonalcoholic fatty liver disease [26] and colitis [27].

**Figure 1:**
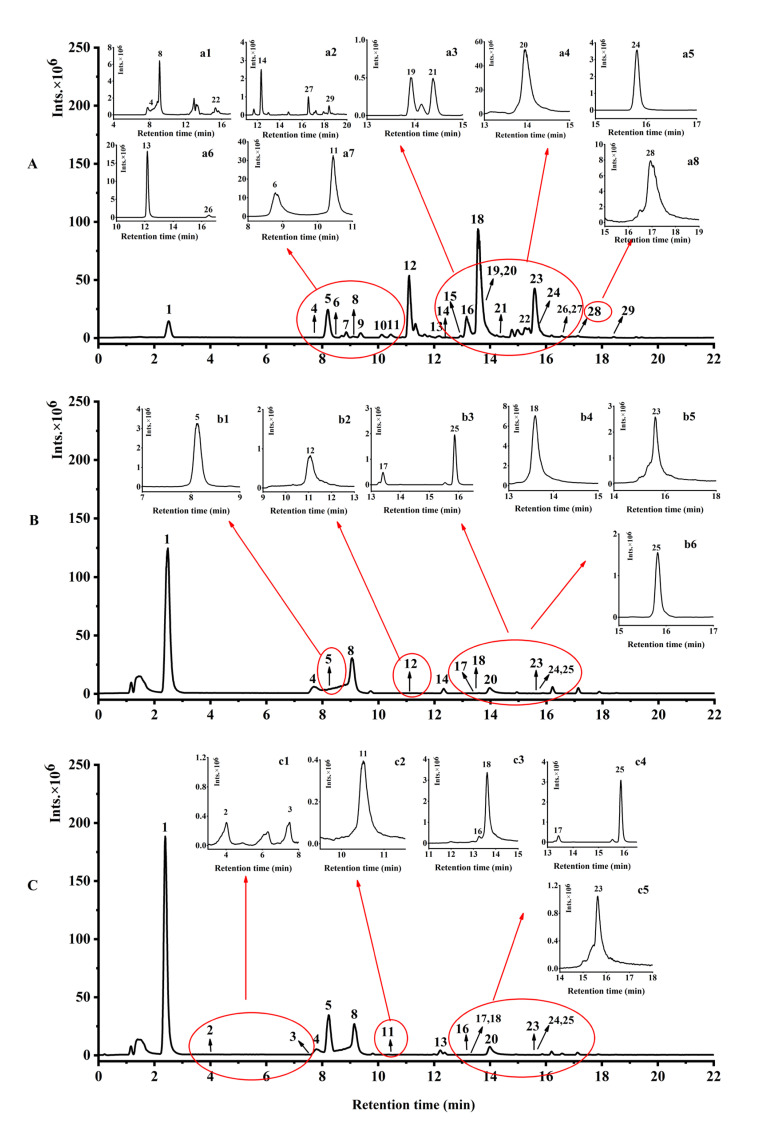
Negative ion current chromatograms of three phenolic fractions from water caltrop (*Trapa quadrispinosa* Roxb.) husk. (**A**–**C**) Represents free phenolic, esterified phenolic and insoluble-bound phenolic fractions, respectively. Panels a1–a8 are the extracted ion chromatograms from (**A**); b1–b6 are the extracted ion chromatograms from (**B**); c1–c5 are the extracted ion chromatograms from (**C**).

Phytochemical compounds of three phenolic fractions from water caltrop husk were comparatively identified by using UHPLC-ESI-HRMS/MS in the negative mode. The ion current chromatograms are illustrated in Figure 1, and the related mass data are summarized in Table 1. Compounds’ identifications were obtained by comparing their mass-related data with those of corresponding authentic standards or results reported earlier. As shown in Figure 1 and Table 1, a total of 29 compounds were tentatively or positively identified as phenolic compounds, including five phenolic acids, two flavonoids, two lignans and 20 gallotannins and their derivatives. For the FP, compounds **5**, **12**, **18** and **23** (peaks **5**, **12**, **18** and **23**, respectively) were found to have the higher peak areas, suggesting that those four phenolics may be the main compounds in the FP extracts. Compound **5** ([M-H]^−^ *m*/*z* = 183.0293) was identified as 4-*O*-methylgallic acid, and the characteristic fragment at *m*/*z* of 140.0140 and 111.0076 [15,28]. Compound **12** ([M-H]^−^ *m*/*z* = 635.0903) was identified as trigalloylglucose and its characteristic fragment ions were 169.0134 and 313.0568 [15,29]. Compound **18** ([M-H]^−^ *m*/*z* = 787.1014) was identified as tetragalloylglucose, and the characteristic fragments at *m*/*z* of 465.0674, 617.0793 and 635.0897 [15,17,29,30]. Compound **23** ([M-H]^−^ m/z = 939.1132) was identified as pentagalloylglucose, and the characteristic fragment ion ([M-H]^−^ m/z = 617.0793) of compound **23** resulted from loss of galloyl moiety 322, indicating the presence of condensed gallic acid as a group on glucose [15,29,30]. However, compound **1** and **8** (peaks **1** and **8**, respectively) were higher peaks in EP and BP. Compound **1** ([M-H]^−^ *m*/*z* = 169.0136) was identified as gallic acid by references, and the characteristic fragment ion ([M-H]^−^ *m*/*z* = 125.0234) of compound **1** was due to the loss of CO_2_ (44Da) [15,30]. Compound **8** ([M-H]^−^ *m*/*z* = 321.0259) was identified as digallic acid by references, and the characteristic fragment ion of 169.0134 [15,30]. Meanwhile, compound **5** (peaks **5**) was the higher peak in BP too. In addition, many other kinds of phenolic compounds were detected in this study. Compounds **13** and **26** were identified as isomers of syringic acid ([M-H]^−^ *m*/*z* = 197.0453), both of which contained fragment ions with an m/z of 123.0074 in their MS^2^ fragment ions, a characteristic ion for syringic acid in the negative ion mode [15,28]. Compound **24** was identified as quercitrin ([M-H]^−^ *m*/*z* = of 447.0941), and the characteristic fragment ion ([M-H]^−^ *m*/*z* = 300.0277) was due to the loss of the rhamnose moiety [17]. Results indicated that gallotannins were abundant in different phenolic extracts of water caltrop husk.

The quantitative results of FP, EP and BP are summarized in Table 1. For the selection of quantitative standards, two rules were followed: If there is a commercial standard, the standard was used for quantification; if not, the standard that can contain the same or similar aglycone for semi-quantification was used. Compounds **1**, **5**, **12**, **16**, **18** and **23** were the most abundant components in FP, accounting for 33.79%, 17.03%, 14.43%, 9.10%, 6.35% and 5.48% of the total identified phenolic compounds in the extract, respectively. Compounds **1** and **8** were the most abundant components in EP, accounting for 78.87% and 14.29% of the total identified phenolic compounds in the extract, respectively. Compounds **1**, **5** and **8** in BP were the most abundant components, which accounted for 74.90%, 15.48% and 5.40% of the total identified phenolic compounds in the extract, respectively. Similar to the results of previous studies, the main phenolic components in different fractions of the same plant material were different [11,17]. In water caltrop husks, the main components of FP were compounds **5**, **12**, **18** and **23**, while compound **1** was the main compound in both EP and BP. The contents of compound **1** in EP and BP were 8.91 times and 11.58 times that in FP, respectively. This may be due to the fact that many insoluble gallotannins bound to plant cell walls or other biomacromolecules were hydrolyzed by strong alkali to produce compound **1** (gallic acid) during the extraction of EP and BP [32].

The total phenolics content was expressed by mg gallic acid equivalents (GAE)/g dry weight of water caltrop husk extract. The content of total flavonoids was expressed by mg rutin equivalents (RE)/g dry weight of water caltrop husk extract. The highest phenolic content in FP was 406.06 ± 3.94 mg GAE/g, while the phenolic contents in EP and BP were 0.68 and 0.88 times that in FP, respectively. The results were consistent with the quantitative results. There was abundant phenolic in water caltrop husk, and it mainly exists in the form of FP. These findings are similar to those reported in previous studies for Chinese sumac (*Rhus chinensis* Mill.) fruits, raspberry pomace and walnut kernel, in which FP contained the highest phenolic content [17,33,34]. The flavonoid contents of FP and EP were similar, 82.85 ± 0.70 mgRE/g and 83.20 ± 0.35 mgRE/g, respectively, without a significant difference (*p* > 0.05). The flavonoid content of BP was significantly higher than that of FP and EP, and the flavonoid content was 99.90 ± 0.35 mgRE/g. Many plants are rich in phenolic compounds, which could be measured by Folin–Ciocalteu reagent method. Compared with those previous reports [11,17], it can be concluded that water caltrop husk is rich in phenolic compounds, which may be a good source of dietary phenolics.

### 3.2. Antioxidant Activity

#### 3.2.1. ABTS Radical Scavenging Activity

**Figure 2:**
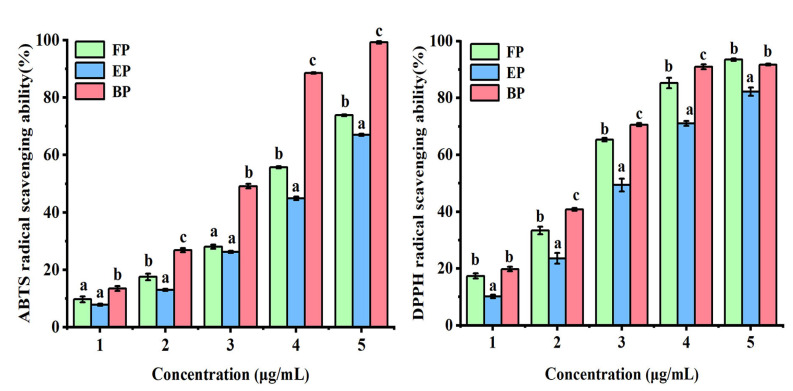
ABTS and DPPH radical scavenging activities of three phenolic fractions from water caltrop (*Trapa quadrispinosa* Roxb.) husk. FP, EP and BP means free phenolic, esterified phenolic and insoluble-bound phenolic fractions. All the values are expressed as mean ± SD (*n* = 3). The different letters at the same concentration means significant differences (*p* < 0.05).

**Figure 3:**
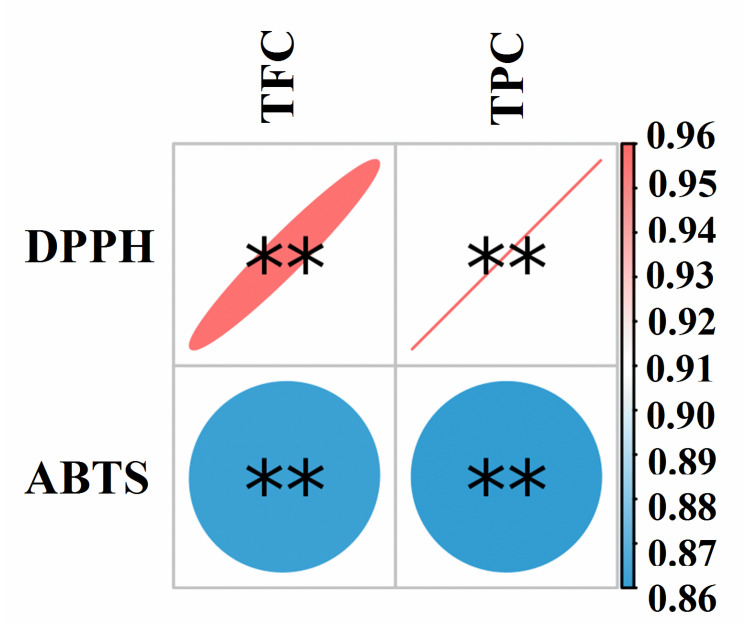
Correlation analyses between total phenolics content (or total flavonoid content) and DPPH radical scavenging activities, ABTS radical scavenging activities of the extracts from water caltrop (*Trapa quadrispinosa* Roxb.) husk. TPC and TFC mean total phenolics content and total flavonoid content, respectively. The legend shows the correlation coefficient value; and blue to red represents an increase in correlation coefficient value from 0.86 to 0.96. * marks “index—factor” with significant correlation, ** stands for *p* < 0.01.

The ABTS radical scavenging ability is one of the commonly used methods to evaluate the antioxidant activity of certain substances [18,35]. Figure 2 shows the scavenging ability of three phenolic extracts in different states of the water caltrop husk against ABTS free radicals. All of the FP, EP, and BP showed good ABTS radical scavenging effects. When the sample concentration was 3.0 μg/mL, the ABTS radical scavenging rates of those three fractions were 73.83%, 67.00% and 99.19%, respectively (*p* < 0.05). The IC_50_ values of FP, EP and BP were 1.86 ± 0.01 μg/mL, 2.14 ± 0.07 μg/mL and 1.15 ± 0.02 μg/mL, respectively. The correlation coefficients of TPC, TFC and the ABTS radical scavenging ability were analyzed with Pearson’s correlation analysis and the results are shown in Figure 3. The results showed that ABTS free radical scavenging capacity was significantly positively correlated with TPC and TFC, indicating that phenolics and flavonoids in water caltrop husk had a significant response to ABTS free radical scavenging capacity (r = 0.86 and 0.86, *p* < 0.01), which was consistent with previous studies [36,37].

#### 3.2.2. DPPH Radical Scavenging Activity

DPPH is a stable free radical, and the evaluation of its scavenging ability is an important indicator to test the antioxidant capacity of natural compounds [18,35]. As shown in Figure 2, FP, EP and BP all showed good DPPH radical scavenging effects. When the concentration was 2.4 μg/mL, the free radical scavenging rates of DPPH were 93.49%, 82.19% and 91.74%, respectively. The IC_50_ values of the FP, EP and BP were 1.04 ± 0.02 μg/mL, 1.34 ± 0.04 μg/mL and 0.82 ± 0.12 μg/mL, respectively (*p* < 0.05). The general trend of DPPH free radical scavenging activities of the phenolic extracts in the three different forms from water caltrop husk were similar to that of ABTS. The correlation coefficients of TPC, TFC and the DPPH free radical scavenging ability by Pearson’s correlation analysis are shown in Figure 3. Results showed that DPPH free radical scavenging rate was also significantly positively correlated with TPC and TFC, indicating that phenolics and flavonoids in water caltrop husk contributed significantly to the DPPH free radical scavenging capacity of those extracts (r = 0.96 and 0.95, *p* < 0.01), which was consistent with the findings reported in previous studies [36,37].

As mentioned above, a large number of studies have shown that elevated levels of oxidative stress can cause cell and tissue damage, thereby resulting in chronic inflammation and metabolic diseases [1,2,3,4,5]. Especially for diabetic patients, long-term high blood glucose level induces elevated oxidative stress and leads to vascular inflammation, which in turn leads to serious complications [7,8,9]. Appropriate dietary antioxidant supplementation can effectively reduce oxidative stress of the body to prevent chronic disease complications [7,8,19]. The present findings showed that the different phenolic extracts of water caltrop husk have strong free radical scavenging abilities; in addition, water caltrop husk is derived from food processing by-products, thus it can be used as a potential source of dietary antioxidants to reduce oxidative stress. However, the in vivo antioxidant effects and metabolic features of those fractions require further studies.

### 3.3. Inhibition of α-Glycosidase

**Figure 4:**
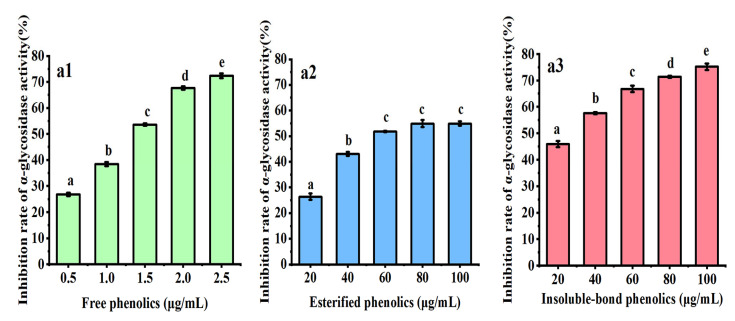
Inhibition rates of three phenolic fractions from water caltrop (*Trapa quadrispinosa* Roxb.) husk towards α-glycosidase. (**a1**–**a3**) represent free phenolics, esterified phenolics, and insoluble-bound phenolics, respectively. All the values are expressed as mean ± SD (*n* = 3). The different letters indicate significant differences (*p* < 0.05).

Hyperglycemia is an important feature of diabetes, which seriously affects human health. α-Glycosidase is a key enzyme for digesting carbohydrate in the intestinal tract. Inhibition of α-glycosidase activity can reduce carbohydrate digestion and absorption, thus delaying postprandial blood glucose rise [20]. Figure 4 shows the inhibitory effects of phenolics in different forms from water caltrop husk towards α-glycosidase. All samples exhibited good inhibitory effects towards α-glycosidase in a dose-dependent manner at all tested concentrations. Among those three fractions, FP showed the strongest inhibitory effect with IC_50_ = 1.43 ± 0.12 μg/mL. Both EP and BP showed weaker inhibitory activity on α-glycosidase than FP, with IC_50_ values of 71.01 ± 1.31 μg/mL and 23.01 ± 0.90 μg/mL, respectively. When the sample concentration reached 100 μg/mL, the α-glycosidase inhibition rate of EP was 54.88%, and the α-glycosidase inhibition rate of BP was 75.24%. The inhibitory effects towards α-glycosidase showed a trend of FP > BP > EP. As shown in Table 1, the trend of TPC content was also displayed as FP > BP > EP, which was consistent with the trend of α-glycosidase inhibition. However, the trend of TFC content was BP > EP > FP, which was inconsistent with the trend of α-glycosidase inhibition. Therefore, it may reasonably conclude that the α-glycosidase inhibitory activity of phenolic extracts was positively correlated with the content of TPC. The phenolic components in different forms may be the main active substances that responded for the α-glycosidase inhibitory activity of water caltrop husk, while the flavonoids’ components may not contribute significantly to this bioactivity. This finding was similar to the finding reported in a previous study about Chinese sumac (*Rhus chinensis* Mill.) fruits [12]. In addition, it was reported that the major contributors to the α-glycosidase inhibitory ability of many other plant extracts may be also phenolics [32,36,38]. In the present work, the IC_50_ value of acarbose (a positive control) was 0.27 ± 0.02 μg/mL (*p* < 0.05).

### 3.4. Molecular Docking

As mentioned above, α-glycosidase can directly participate in the metabolism of starch and glycogen to hydrolyze glycosidic bonds and release glucose, which is an indispensable enzyme in human glucose metabolism [9,12]. Its activity is positively correlated with postprandial blood glucose level. Therefore, inhibition of α-glycosidase activity can effectively reduce or delay postprandial blood glucose. The purpose of this study was to investigate the interaction of phenolics from water caltrop husk and α-glycosidase by molecular docking method. The molecular docking results are summarized in Table 1 and Table 2. The absolute value of the affinity energy of trigalloylglucose and tetragalloylglucose was greater than 10, which indicates that they bind to α-glycosidase well. Therefore, three different trigalloylglucose isomerides and three different tetragalloylglucose isomerides that were reported were selected for molecular docking analysis in this study.

**Figure 5:**
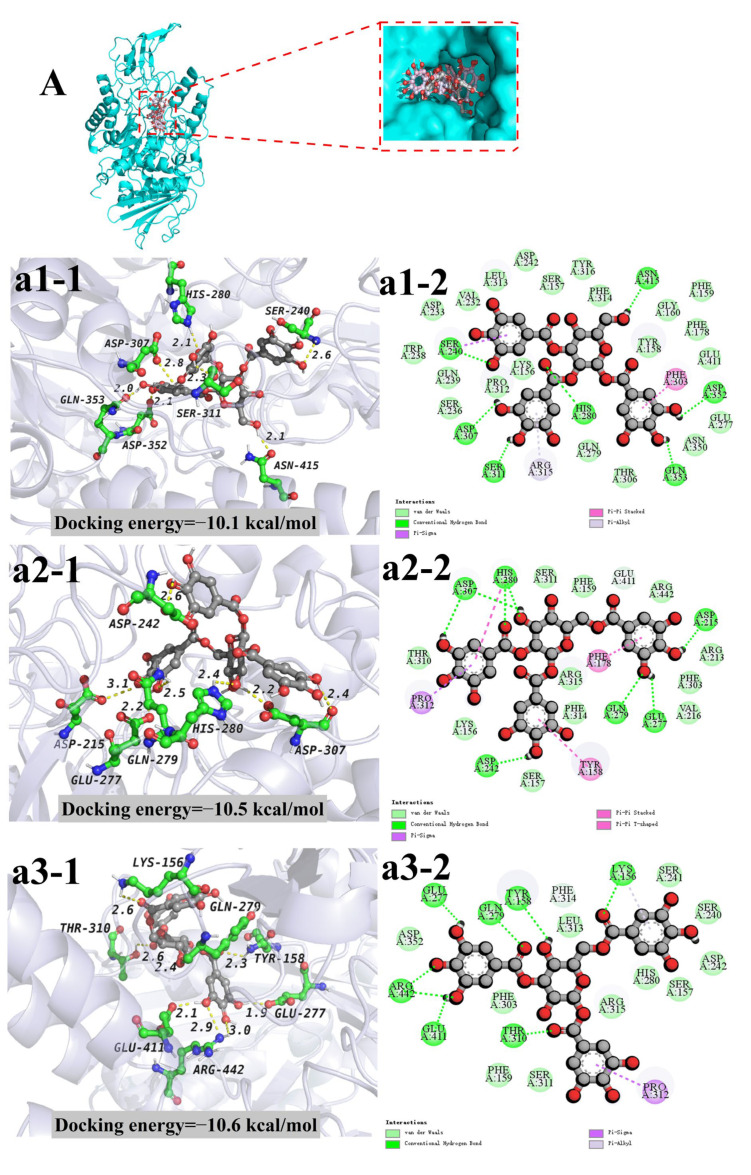

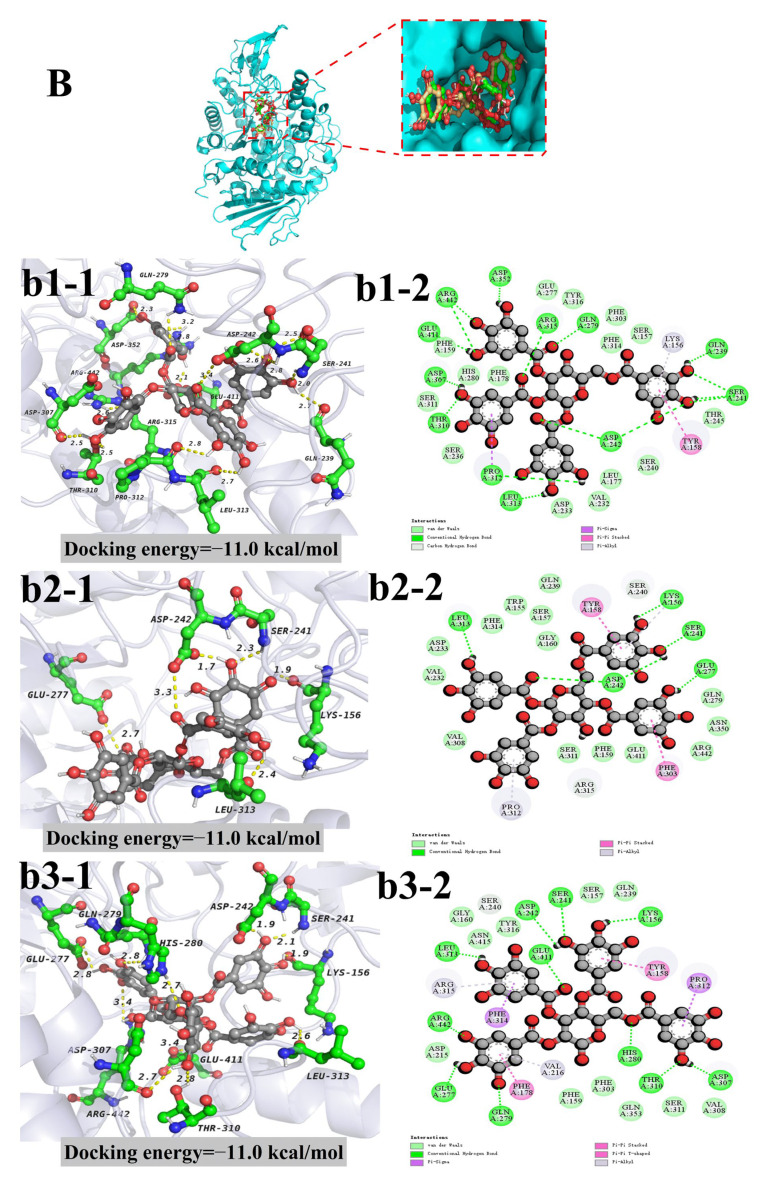
Molecular docking results of three different structures of trigalloylglucose (**A**) and three different structures of tetragalloylglucose (**B**) with α-glycosidase complexes. (**A**,**B**) The pocket positions of the three compounds that bind to α-glycosidase. (**a1**–**a3**) Hydrogen bonding and hydrophobic interactions formed by 1,2,3-trigalloylglucose (**a1**), 1,2,6-trigalloylglucose (**a2**) and 1,3,6-trigalloylglucose (**a3**) with α-glycosidase conformation diagram. Additionally, (**b1**–**b3**) hydrogen bonding and hydrophobic interactions formed by 1,2,4,6-tetragalloylglucose (**b1**), 1,2,3,6-tetragalloylglucose (**b2**) and 2,3,4,6-tetragalloylglucos (**b3**) with α-glycosidase conformation diagram. The (**a1-1**–**a3-1**, **b1-1**–**b3-1**) represent the 3D conformation of the hydrogen bond, while (**a1-2**, **a2-2**, **a3-2**, **b1-2**, **b2-2**, **b3-2**) represent the 2D conformation of the hydrogen bond with hydrophobic interactions, respectively.

As shown in Figure 5 and Table 3, the 1,2,3-trigalloylglucose formed seven hydrogen bonds with seven amino acid residues at the active site of α-glycosidase, and the longest and shortest hydrogen bond distances are 2.8 Å and 2.0 Å, respectively, with an average hydrogen bond distance of 2.29 Å. The 1,2,6-trigalloylglucose mainly forms six hydrogen bonds with six amino acid residues at the active site, and the longest and the shortest distances of hydrogen bond in the hydrogen bonding were 3.1 Å and 2.2 Å, the average hydrogen bond distance is 2.48 Å. The 1,3,6-trigalloylglucose mainly forms eight hydrogen bonds with seven amino acid residues at the active site, and the longest and shortest hydrogen bond distances are 3.0 Å and 1.9 Å, respectively, with an average hydrogen bond distance of 2.47 Å. The 1,2,3,6-tetragalloylglucose formed 15 hydrogen bonds with 12 amino acid residues at the active site of α-glycosidase, and the longest and shortest hydrogen bond distances are 3.4 Å and 2.0 Å, respectively, with an average hydrogen bond distance of 2.63 Å. The 1,2,4,6-tetragalloylglucose formed six hydrogen bonds with five amino acid residues at the active site of α-glycosidase, and the longest and shortest hydrogen bond distances are 3.3 Å and 1.7 Å, respectively, with an average hydrogen bond distance of 2.38 Å. The 2,3,4,6-tetragalloylglucose formed 11 hydrogen bonds with 11 amino acid residues at the active site of α-glycosidase, and the longest and shortest hydrogen bond distances are 3.4 Å and 1.9 Å, respectively, with an average hydrogen bond distance of 2.64 Å. Previous studies have reported that the number and distance of hydrogen bonds are the main driving factors for the interaction between the docking ligand and a-glycosidase; the ligand binds to the active amino acid of α-glycosidase through the hydrogen bond to prevent the substrate from entering, thereby inhibiting α-glycosidase activity [10,39,40]. Among three different trigalloylglucose isomerides, the average hydrogen bond of the complex formed between 1,2,3-trigalloylglucose and α-glycosidase was shorter, but the number of hydrogen bonds and the number of amino acid residues of the complex formed by the three kinds of trigalloylglucose and α-glycosidase were very close. The average bond length of 1,2,3,6-tetragalloylglucose was the longer, but it had the highest number of hydrogen bonds. The complex that formed 1,2,4,6-tetragalloylglucose and α-glycosidase had the shortest average bond length but the lowest number of hydrogen bonds. Moreover, previous studies have shown that Glu-277 plays a key role in the catalytic site of α-glycosidase [10,39]. According to the docking results, 1,2,6-trigalloylglucose, 1,3,6-trigalloylglucose, 1,2,4,6-tetragalloylglucose and 2,3,4,6-tetragalloylglucos formed hydrogen bonds with Glu-277. In both 1,2,6-trigalloylglucose and α-glycosidase complex and 1,3,6-trigalloylglucose and α-glycosidase complex, the hydrogen bonds formed with Glu-277 were all the shortest in the complex, indicating that 1,2,6-trigalloylglucose and 1,3,6-trigalloylglucose may have the strongest interaction with Glu-277. In addition, it has been reported that Asp-242 also plays a crucial role in the catalytic site of α-glycosidase [40]. In the current work, it is found that both 1,2,4,6-Tetragalloylglucosglucose and 2,3,4,6-Tetragalloylglucos formed a hydrogen bond with Asp-242, and this hydrogen bond was the shortest, indicating that both the 1,2,4,6-tetragalloylglucglucose and 2,3,4,6-tetragalloylglucos may have the strongest interaction with Asp-242. Meanwhile, it has been also found that Ser-241 plays a crucial role in the catalytic site of α-glycosidase [41], and 1,2,4,6-tetragalloylglucose, 1,2,3,6-tetragalloylglucose and 2,3,4,6-tetragalloylglucos all formed hydrogen bonds with Ser-241. The hydrogen bond formed with Ser-241 was the shortest in the 1,2,3,6-tetragalloylglucose and α-glycosidase complex, indicating that 1,2,3,6-tetragalloylglucose may have the strongest interaction with Ser-241. Glu411, one of the residues that interacted with 1,3,6-trigalloylglucose, 1,2,3,6-tetragalloylglucose and 2,3,4,6-tetragalloylglucos, has also been proved to play a key role in the catalytic site of α-glycosidase [9,42]. In conclusion, the docking analysis results revealed that all of those compounds could interact well with the key amino acid residues of α-glycosidase. However, only with docking analysis can which compound formed the most stable complex with α-glycosidase not be judged clearly. Therefore, the structural mechanisms of their interactions will be further investigated in the following molecular dynamics.

### 3.5. Molecular Dynamic Study

This study used 100-ns molecular dynamics simulations to further investigate the stability of the trigalloylglucose and α-glycosidase complex or tetragalloylglucose and α-glycosidase complex. The α-glycosidase was used as a control protein. Ultimately, the differences between those compound-enzyme complexes with different conformations were investigated.

**Figure 6:**
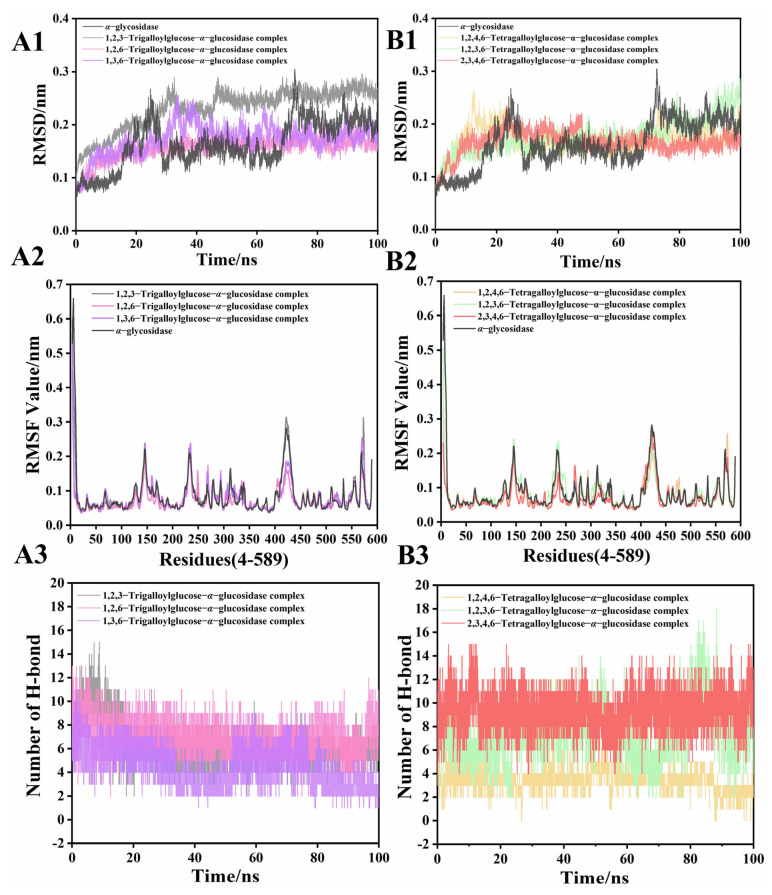
Molecular dynamics (100 ns) results of three different structures of trigalloylglucose and α-glycosidase complexes (**A**) or three different structures of tetragalloylglucose and α-glycosidase complexes (**B**). (**A1**,**B1**) represent the root mean square deviations (RMSD, nm). (**A2**,**B2**) represent the root mean square fluctuations (RMSF, nm). (**A3**,**B3**) represent the number of hydrogen bonds (H-bonds, number) of three different complexes and α-glycosidase alone at 100 ns molecular kinetics, respectively.

The RMSD was used to determine the average deviation between the conformation and initial conformation of the complex in the system at the specific time, and it was generally used to evaluate whether the complex had reached a stable state in this system [41]. Figure 6A1 shows the RMSD results for the three isomers of trigalloylglucose in complex with α-glycosidase. Results clearly show that the 1,2,6-trigalloylglucose and α-glycosidase complex (pink) is stable at 30 ns and has an RMSD value of 0.17 nm. Additionally, the stability and RMSD value of the other complexes are all weaker. Figure 6B1 shows the RMSD results for three isomers of tetragalloylglucose in complex with α-glycosidase. Results clearly show that the 2,3,4,6-tetragalloylglucose and α-glycosidase complex (red) reached stability at 50 ns and had an RMSD value of 0.17 nm. Meanwhile, the other two complexes did not reach a steady state in the 100 ns molecular dynamics simulations. The fluctuations in the RMSD values of α-glycosidase exceeded those of all complexes in the 100 ns molecular dynamics simulations. Thus, it is suggested that both the above trigalloylglucose and tetragalloylglucose can bind to α-glycosidase to form stable complexes.

The RMSF was used to analyze the fluctuation of the receptors’ residues by their reference position during the simulation period [41]. As shown in Figure 6A2,B2, the overall fluctuation of amino acid residues of α-glycosidase is significantly stronger than that of the complex. Therefore, it indicated that trigalloylglucose or tetragalloylglucose combines with α-glycosidase to form a complex, which will lead to a tighter structure of the protein. The molecular docking results show that amino acid residues located in the active pocket of α-glycosidase formed hydrogen bonds, van der Waals force, hydrophobic interaction and other weak interaction forces with trigalloylglucose or tetragalloylglucose. In comparison with α-glycosidase (blank), the combining complex of the enzyme and 1,2,6-trigalloylglucose or 2,3,4,6-tetragalloylglucose showed overall significantly smaller fluctuations of amino acid residues in the binding region (between Ala200 and Gln450). In molecular docking, the following four key amino acids were involved in most complexes: Ser-241, Asp-242, Glu-277 and Glu-411. Glu-277 and Glu-411 of α-glycosidase have been reported in many studies to form hydrogen bonds with ligands to make the complex more stable [9,43]. In the present study, the RMSF values of the ligand-enzyme complexes at both Glu-277 and Glu-411 were relatively small, which also suggested that the ligands interact with Glu-277 and Glu-411 to make the complex more stable. It is worth noting that previous studies have reported that the galloyl moiety was able to enter the active pocket of α-glycosidase, creating weak interaction forces such as hydrogen bonds and hydrophobic interactions with amino acid residues in the active site [42]. Therefore, the gallic acid may interact with the active amino acid residues of α-glycosidase (e.g., Glu2-77 and Glu-411), leading to a reduction of α-glycosidase activity. As shown in Figure 6A3,B3, hydrogen bonds always exist between trigalloylglucose and α-glycosidase or tetragalloylglucose and α-glycosidase in 100-ns molecular dynamics simulations. Although the number of hydrogen bonds fluctuates, hydrogen bonds exist moderately. In the 1,2,6-trigalloylglucose and α-glycosidase complex (Figure 6A3), the number of hydrogen bonds fluctuates around seven, while, in the 2,3,4,6-tetragalloylglucose and α-glycosidase complex (Figure 6B3), the number of hydrogen bonds fluctuates around 10. Tetragalloylglucose has one more galloyl moiety than trigalloylglucose, and studies have shown that the galloyl moiety can facilitate the formation of hydrogen bonds with amino acid residues of α-glycosidase [42,44]. However, in molecular dynamics simulations, the current study found that 1,2,6-trigalloylglucose could form a more stable complex with α-glycosidase when compared to 2,3,4,6-tetragalloylglucose (as shown by RMSD fluctuations), which may be due in part to steric hindrance. Meanwhile, hydrophobic interaction forces were also previously reported to be a key force in maintaining the stability of ligands and proteins [9,40]. The aforementioned results indicated that both 1,2,6-trigalloylglucose and 2,3,4,6-tetragalloylglucose may have better α-glycosidase inhibition than their own other two isomers, and moreover, the 1,2,6-trigalloylglucose may possess stronger inhibition than 2,3,4,6-tetragalloylglucose.

## 4. Conclusions

In this study, a total of 29 substances were identified and quantified. Tannins were the main constituents of water caltrop husk extract. They mainly existed in the form of trigalloylglucose, tetragalloylglucose and pentagalloylglucose. All of the free phenolic (FP), esterified phenolic (EP) and insoluble-bound phenolic (BP) fractions exhibited good antioxidant activities, and the BP had the highest radical scavenging ability with IC_50_ values of 0.82 ± 0.12 μg/mL (ABTS) and 1.15 ± 0.02 μg/mL (DPPH), followed by the FP whose IC_50_ values were 1.86 ± 0.01 μg/mL (ABTS) and 1.04 ± 0.02 μg/mL (DPPH). Additionally, the IC_50_ values for the antioxidant activity of EP were 2.14 ± 0.07 μg/mL (ABTS) and 1.34 ± 0.04 μg/mL (DPPH). Pearson correlation analysis showed that TPC and TFC had a significant positive correlation with the two in vitro antioxidant activities. When compared with the EP and BP, the FP showed the strongest inhibition towards the α-glycosidase and the IC_50_ value of FP was 1.43 ± 0.12 μg/mL, and the IC_50_ values of EP and BP inhibiting α-glycosidase were 71.01 ± 1.31 μg/mL and 23.01 ± 0.90 μg/mL, respectively. By molecular docking and molecular dynamics analysis, all three isomers of trigalloylglucose or tetragalloylglucose can combine with α-glycosidase through hydrogen bonds and hydrophobic interaction forces to form stable complexes. Among them, the complex formed by 1,2,6-trigalloylglucose and α-glycosidase was the most stable. Although molecular dynamics is currently becoming a powerful and flexible investigative tool in the most diverse fields of structural biology and materials science, further studies should be conducted in the future to evaluate the effects of water caltrop husk extracts even in vivo. In sum, this study may provide a potential natural resource for the improvement of oxidative stress injury and blood glucose control in diabetes mellitus, and expand the use of water caltrop husk to improve its economic value.

## Figures and Tables

**Table 1:** The identified phenolic compounds, total phenolics and total flavonoids in the free, esterified and insoluble-bound phenolic fractions from water caltrop (*Trapa quadrispinosa* Roxb.) husk.

Peak	Compounds	TR * (min.)	[M-H]^−^ (m/z)	Molecular Formula	MS/MS Fragment Ions	Reference	Extracts		
							FP	EP	BP
1	Gallic acid	2.51	169.0136	C_7_H_5_O_5_	125.0234 (100.00), 69.0332 (98.97)	[15]	3364.65 ± 13.67 ^c^	30,001.66 ± 175.78 ^b^	38,947.59 ± 56.68 ^a^
2	Di-galloylglucose isomer I	4.00	483.0793	C_20_H_19_O_14_	169.0134 (100.00), 125.0235 (15.81)	[15,30]	ND	ND	4.73 ± 0.24
3	Di-galloylglucose isomer II	7.51	483.0794	C_20_H_19_O_14_	169.0134 (100.00), 211.0244 (28.64)	[15,30]	ND	ND	8.39 ± 0.42
4	Digallic acid isomer I	7.81	321.0259	C_14_H_9_O_9_	125.0232 (100.00), 169.0134 (71.97)	[15,30]	20.37 ± 1.02 ^c^	605.37 ± 30.27 ^a^	395.43 ± 19.77 ^b^
5	4-*O*-Methylgallic acid	8.21	183.0293	C_8_H_7_O_5_	140.0140 (100.00) 111.0076 (64.41), 139.0027 (58.57)	[15,28]	5589.97 ± 55.89 ^b^	70.15 ± 3.51 ^c^	8050.32 ± 17.62 ^a^
6	DiG-HHDP-glucose isomer I	8.74	785.0866	C_34_H_25_O_22_	300.9990 (82.14)	[15]	274.57 ± 8.24	ND	ND
7	Di-galloylglucose isomer III	8.85	483.0792	C_20_H_19_O_14_	271.0461 (100.00) 169.0134 (85.86), 211.0244 (24.05)	[15,30]	1044.93 ± 10.45	ND	ND
8	Digallic acid isomer II	9.04	321.0259	C_14_H_9_O_9_	125.0233 (100.00), 169.0134 (71.99)	[15,30]	111.76 ± 5.59 ^c^	5366.87 ± 162.18 ^a^	2816.03 ± 140.80 ^b^
9	Trigalloylglucose isomer I	9.35	635.0909	C_27_H_23_O_18_	169.0134 (100.00), 313.0568 (62.85)	[15,29]	919.29 ± 45.96	ND	ND
10	Trigalloylglucose isomer II	10.12	635.0906	C_27_H_23_O_18_	169.0134 (59.59), 313.0566 (42.80)	[15,29]	617.04 ± 8.02	ND	ND
11	DiG-HHDP-glucose isomer II	10.45	785.0863	C_34_H_25_O_22_	300.9991 (100.00)	[15]	664.41 ± 13.29 ^a^	ND	7.82 ± 0.39 ^b^
12	Trigalloylglucose isomer III	11.11	635.0903	C_27_H_23_O_18_	313.0568 (23.98) 169.0134 (21.22),	[15,29]	10,460.24 ± 102.58 ^a^	20.85 ± 1.04 ^b^	ND
13	Syringic acid isomer I	12.14	197.0453	C_9_H_9_O_5_	123.0074 (5.67)	[28]	383.80 ± 10.48 ^b^	ND	590.59 ± 3.51 ^a^
14	*p*-Coumaric acid isomer I	12.34	163.0394	C_9_H_7_O_3_	119.0490 (100.00), 93.0368 (25.31)	[30]	51.09 ± 2.04 ^b^	364.83 ± 18.24 ^a^	ND
15	Tri-*O*-galloyl-HHDP-D-glucose	13.05	937.0975	C_41_H_29_O_26_	169.0130 (1.90), 767.0746 (4.85)	[15]	2886.32 ± 13.54	ND	ND
16	Tetragalloylglucose isomer I	13.17	787.1012	C_34_H_27_O_22_	465.0674 (100.00), 635.0897 (47.64)	[15,17,29,30]	3898.93 ± 129.39 ^a^	ND	3.19 ± 0.16 ^b^
17	Isolariciresinol 9-*O*-glucoside isomer I	13.41	521.2041	C_26_H_33_O_11_	101.0231 (24.47), 89.0231 (21.59)	[20]	ND	trace	trace
18	Tetragalloyl glucose isomer II	13.56	787.1014	C_34_H_27_O_22_	617.0793 (100.00) 465.0676 (44.87)	[15,17,29,30]	20,762.27 ± 510.50 ^a^	126.28 ± 6.31 ^b^	62.61 ± 3.76 ^c^
19	Myricitrin	13.90	463.0894	C_21_H_19_O_12_	316.0225 (100.00), 317.0282 (33.49)	Standard	15.67 ± 0.78	ND	ND
20	Ellagic acid	13.95	300.9994	C_14_H_5_O_8_	283.9969 (20.92), 201.0186 (14.63)	Standard	963.22 ± 31.75 ^b^	931.76 ± 37.93 ^c^	1442.99 ± 104.08 ^a^
21	Tri-*O*-galloyl-brevifolincarboxyl-D-glucose	14.37	909.1033	C_40_H_29_O_25_	435.0587 (15.78), 291.0146 (13.30)	[15]	78.99 ± 3.95	ND	ND
22	Digallic acid isomer III	15.29	321.0253	C_14_H_9_O_9_	125.0232 (100.00), 169.0134 (57.66)	[15,30]	14.19 ± 0.71	ND	ND
23	Pentagalloylglucose	15.59	939.1132	C_41_H_31_O_26_	787.1010 (100.00), 617.0793 (12.54)	[15,29,30]	8868.45 ± 86.43 ^a^	41.45 ± 2.07 ^b^	18.69 ± 0.46 ^b^
24	Quercitrin	15.82	447.0941	C_21_H_19_O_11_	301.0345 (74.98), 300.0277 (100.00)	[31]	38.55 ± 1.93 ^a^	19.62 ± 0.98 ^b^	18.06 ± 0.66 ^b^
25	Isolariciresinol 9-*O*-glucoside isomer II	15.87	521.2043	C_26_H_33_O_11_	89.0231 (100.00), 101.0231 (37.42)	[20]	ND	12.40 ± 0.03 ^b^	27.97 ± 0.39 ^a^
26	Syringic acid isomer II	16.43	197.0452	C_9_H_9_O_5_	123.0075 (1.97)	[28]	142.24 ± 7.34	ND	ND
27	*p*-Coumaric acid isomer II	16.54	163.0393	C_9_H_7_O_3_	93.0332 (18.45), 119.0492 (3.94)	[30]	18.56 ± 0.93	ND	ND
28	Hexagallyol glucose	16.95	1091.1245	C_48_H_35_O_30_	617.0792 (1.89), 939.1115 (80.16)	[15]	236.72 ± 11.84	ND	ND
29	*p*-Coumaric acid isomer III	18.38	163.0394	C_9_H_7_O_3_	93.0333 (19.65), 119.0490 (52.81)	[30]	11.85 ± 1.06	ND	ND
	TPC (mgGAE/g)						406.06 ± 3.94 ^a^	275.96 ± 2.34 ^b^	360.06 ± 3.94 ^c^
	TFC (mgRE/g)						82.85 ± 0.70 ^b^	83.20 ± 0.35 ^b^	99.90 ± 0.35 ^a^

* TR: retention time; FP, EP and BP mean free phenolic, esterified phenolic and insoluble-bound phenolic fractions, respectively. TPC: total phenolics content; TFC: total flavonoids content. Compounds **17** and **25** were semi-quantified with hesperidin standard; compounds **13** and **26** were quantified with syringic acid standard; compounds **14**, **27** and **29** were quantified with *p*-coumaric acid standard; compound **19** was quantified with myricitrin standard; compound **20** was quantified with ellagic acid standard; compound **24** was quantified with quercetin standard and the remaining compounds were quantified or semi-quantified with gallic acid standard. All the values are expressed as mean ± SD (*n* = 3) with μg/g of dry extract. Lowercase letters (a, b, c) on the same line indicate a significant difference (*p* < 0.05).

**Table 2:** The virtual screening results of mainly identified phenolic compounds from water caltrop (*Trapa quadrispinosa* Roxb.) husk.

Compounds	Pubchem ID	Docking Energy (kcal/mol)	Compounds	Pubchem ID	Docking Energy (kcal/mol)
2,3,4,6-Tetragalloylglucose	49,777,225	−11.0	2,3,6-Trigalloylglucose	129,819,625	−8.9
1,2,4,6-Tetragalloylglucose	11,297,287	−11.0	Quercitin	5,280,459	−8.8
1,2,3,6-Tetragalloylglucose	73,178	−11.0	Myricitin	5,281,673	−8.7
1,3,6-Trigalloylglucose	452,707	−10.6	1,2,3,4-Tetragalloylglucose	133,556,532	−8.5
1,2,6-Trigalloylglucose	440,308	−10.5	Digallic acid	341	−8.5
1,2,3-Trigalloylglucose	13,270,010	−10.1	Ellagic acid	5,281,855	−8.3
Pentagalloylglucose	65,238	−10.0	*p*-Coumaric acid	637,542	−6.7
1,2,6-Trigalloylglucose	124,156,722	−9.8	Methylgallate	7428	−6.5
(-)-Isolariciresinol 9-*O*-glucoside	74,191,750	−9.7	Gallic acid	370	−6.4
(+)-Isolariciresinol 9-*O*-glucoside	93,473,218	−9.6	4-*O*-Methylgallic acid	78,016	−6.5
DiG-HHDP-glucose	471,120	−9.2	Syringic acid	10,742	−5.7

**Table 3:** The molecular docking results of 1,2,3-trigalloylglucose, 1,2,6-trigalloylglucose, 1,3,6-trigalloylglucose, 1,2,3,6-tetragalloylglucose, 1,2,4,6-tetragalloylglucose and 2,3,4,6-tetragalloylglucose with α-glycosidase.

Compounds	Pubchem ID	Docking Energy (kcal/mol)	The Number of Amino Acid Residues Forming Van der Waals Force	Amino Acid Residues Forming Hydrogen Bonds (Bond Length)
1,2,3-Trigalloylglucose	132,707	−10.1	21	His-280 (2.1 Å), Asp-307 (2.8 Å), Gln-353 (2.0 Å) Asp-352 (2.1 Å), Ser-311 (2.3 Å), Asn-415 (2.1 Å), Ser-240 (2.6 Å)
1,2,6-Trigalloylglucose	440,308	−10.5	10	Asp-242 (2.6 Å), Asp-215 (3.1 Å), Glu-277 (2.2 Å), Gln-279 (2.5 Å), His-280 (2.4 Å), Asp-307 (2.4 Å)
1,3,6-Trigalloylglucose	452,707	−10.6	11	Lys-156 (2.6 Å), Thr-310 (2.6 Å), Gln-279 (2.4 Å), Tyr-158 (2.3 Å), Glu-411 (2.1 Å), Glu-277 (1.9 Å), Arg-442 (2.9 Å,3.0 Å)
1,2,3,6-Tetragalloylglucose	73,178	−11.0	15	Asp-352 (2.3 Å), Arg-442 (2.6 Å,3.2 Å), Glu-411 (3.4 Å), Asp-307 (2.5 Å), Thr-310 (2.5 Å), Pro-312 (2.8 Å), Leu-313 (2.7 Å), Asp-242 (2.6 Å), Arg-315 (2.8 Å) Ser-241 (2.5 Å, 2.8 Å, 2.0 Å), Gln-239 (2.7 Å), Gln-279 (2.8 Å)
1,2,4,6-Tetragalloylglucose	11,297,287	−11.0	14	Glu-277 (2.7 Å), Asp-242 (3.3 Å, 1.7 Å), Ser-241 (2.3 Å), Lys-156 (1.9 Å), Leu-313 (2.4 Å)
2,3,4,6-Tetragalloylglucose	49,777,225	−11.0	11	Glu-277 (2.8 Å), Gln-279 (2.8 Å), His-280 (2.7 Å), Thr-310 (2.8 Å), Asp-307 (2.7 Å), Lys-156 (1.9 Å), Ser-241 (2.1 Å), Asp-242 (1.9 Å), Glu-411(3.4 Å), Leu-313 (2.6 Å), Arg-442 (3.4 Å)

## Data Availability

The data that support the findings of this study are available from the corresponding author upon reasonable request.
